# A hybrid computational approach for efficient Alzheimer’s disease classification based on heterogeneous data

**DOI:** 10.1038/s41598-018-27997-8

**Published:** 2018-06-27

**Authors:** Xuemei Ding, Magda Bucholc, Haiying Wang, David H. Glass, Hui Wang, Dave H. Clarke, Anthony John Bjourson, Le Roy C. Dowey, Maurice O’Kane, Girijesh Prasad, Liam Maguire, KongFatt Wong-Lin

**Affiliations:** 1 Intelligent Systems Research Centre, Ulster University, Magee Campus, Derry~Londonderry, Northern Ireland UK; 2 Faculty of Mathematics and Informatics, Fujian Normal University, Fuzhou, China; 3 School of Computing and Mathematics, Ulster University, Jordanstown Campus, Northern Ireland UK; 4 Clarke Analytics Ltd., 6 Dernville, Annabella Mallow, Cork, Ireland; 5 Northern Ireland Centre for Stratified Medicine, Biomedical Sciences Research Institute, C-TRIC, Ulster University, Altnagelvin Hospital, Derry~Londonderry, Northern Ireland UK; 6 C-TRIC, Altnagelvin Hospital campus, Derry~Londonderry, Northern Ireland UK; 7 School of Biomedical Sciences, Ulster University, Coleraine Campus, Northern Ireland UK

## Abstract

There is currently a lack of an efficient, objective and systemic approach towards the classification of Alzheimer’s disease (AD), due to its complex etiology and pathogenesis. As AD is inherently dynamic, it is also not clear how the relationships among AD indicators vary over time. To address these issues, we propose a hybrid computational approach for AD classification and evaluate it on the heterogeneous longitudinal AIBL dataset. Specifically, using clinical dementia rating as an index of AD severity, the most important indicators (mini-mental state examination, logical memory recall, grey matter and cerebrospinal volumes from MRI and active voxels from PiB-PET brain scans, ApoE, and age) can be automatically identified from parallel data mining algorithms. In this work, Bayesian network modelling across different time points is used to identify and visualize time-varying relationships among the significant features, and importantly, in an efficient way using only coarse-grained data. Crucially, our approach suggests key data features and their appropriate combinations that are relevant for AD severity classification with high accuracy. Overall, our study provides insights into AD developments and demonstrates the potential of our approach in supporting efficient AD diagnosis.

## Introduction

Alzheimer’s disease (AD) is a devastating neurodegenerative disorder with incompletely characterised etiology and no effective treatment at present. AD and its prodromal stage, mild cognitive impairment (MCI), are associated with multiple pathogenesis, markers and risks. For example, age is a well-known risk indicator of developing AD^1^. Medical history of neurological disorder has also been linked to higher AD risk^2^. In terms of biomarkers, apolipoprotein E (ApoE) ε4 allele is associated with higher AD risk than the more common ApoE ε3 allele^3,4^. ApoE ε4 allele has also been linked to two key pathologies: beta amyloid, a major component of senile plaque of AD, and the hyperphosphorylation of microtubule-associated tau protein that leads to neurofibrillary tangles^5–7^. Brain imaging data such as magnetic resonance imaging (MRI) and positron-electron tomography (PET) with [^11^C]-Pittsburgh compound B (PiB) or [^18^F]-fluorodeoxyglucose (FDG) tracers have been shown to be a reliable source of information for the diagnosis and prediction of AD progression with accuracy (area under receiver operating characteristic curve, AUC^8^) being 0.82 and 0.87 in^9,10^ respectively. Some other studies focusing on brain imaging data were conducted for AD identification, such as^11,12^. Within clinical settings, psychological/functional assessments are typically conducted, which may include mini-mental state examination (MMSE), logical memory immediate/delayed recall assessments (LMIR/LMDR), and clinical dementia rating (CDR)^13^.

Thus, it is clear that the disease can be linked to factors across multiple domains^14^. Further, our recent work on a multimodal kernel approach applied to combined MRI-PET neuroimaging data has shown more accurate AD diagnosis and prognosis than each individual modality^15^. Thus, multimodal data fusion may provide a more accurate and holistic picture of AD data, and better decision support for AD diagnosis and prognosis^16^. In addition to the aforementioned brain imaging multimodalities, one may also augment these with psychological/functional assessments, blood tests, ApoE genotype, and medical history. However, developing a systematic and automated analytical approach for such heterogeneous AD data is still an open issue. Despite extensive studies on AD, most of studies considered only a limited number of factors, potentially insufficient to provide a systemic understanding or characterization of this complex disease. For example, a novel multiple kernel learning framework combing multimodal features for AD classification was proposed in^17^, however only imaging data, i.e. cerebrospinal fluid biomarkers (CSF) and MRI, were taken into account. In addition, the analysis was based on the limited data set including 70 healthy controls and 50 progressive MCI patients. A hybrid model, combining a feature reduction technique using rough sets and a genetic algorithm and an uncertain reasoning technique based on Bayesian networks (BN), was proposed in^18^, but only psychological/functional assessments were conducted and the obtained BN did not show the strength of the corresponding relationships among the assessments, nor the evolution of the BNs across time.

Therefore, it is still not completely known what factors are relatively more important than others with respect to AD, and how they can be influenced under certain conditions or stages of the disease. With data related to AD becoming more readily available, mathematical and computational approaches become necessary to integrate, analyse and visualise large, complex, and heterogeneous data to provide holistic insights into the disease mechanisms, improve diagnosis and risk predictions, and suggest stratified treatments or interventions^19,20^. However, a systemic computational approach that can rapidly integrate coarse-grained, heterogeneous data for AD classification is currently lacking.

**Figure 1:**
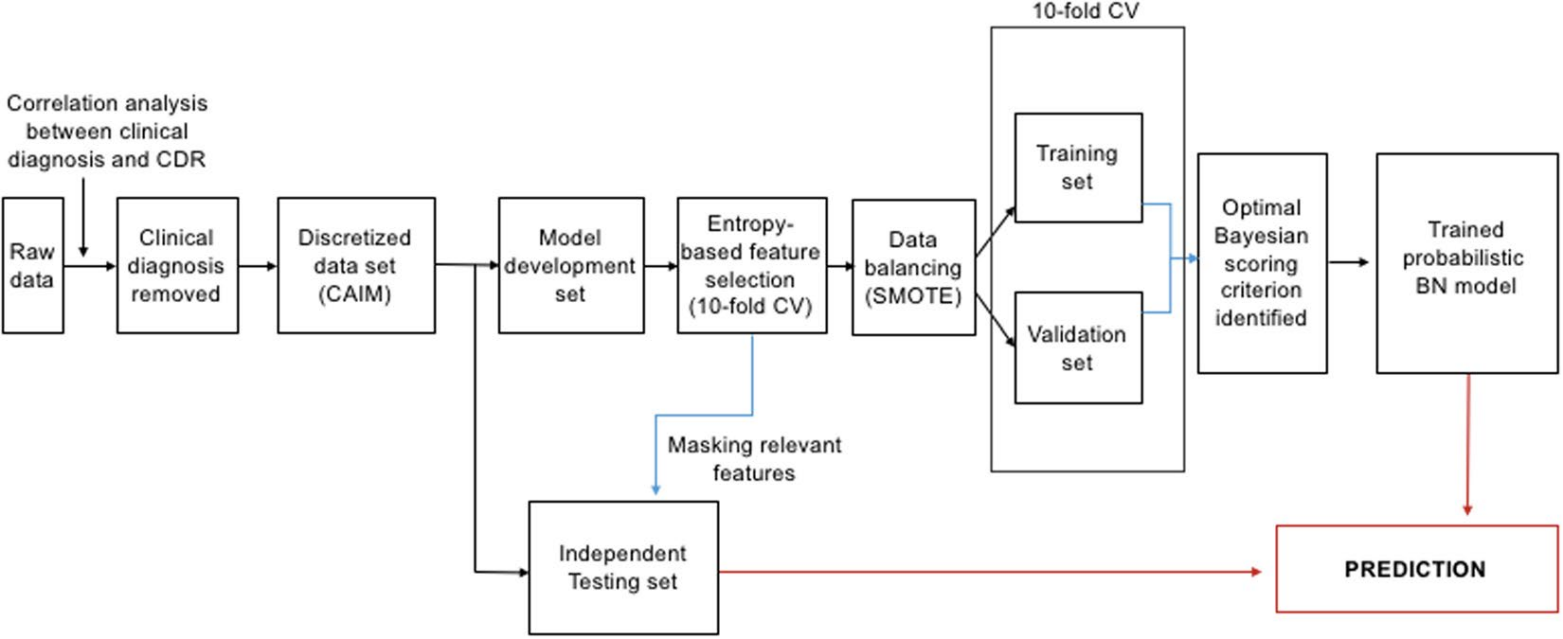
Proposed hybrid computational framework. CAIM: class-attribute interdependence maximization. 10-fold CV: 10-fold cross validation. SMOTE: synthetic minority over-sampling technique. Correlation analysis validates usage of clinical dementia rating (CDR) as an index of AD severity. The CAIM algorithm is used to discretize the considered features with respect to CDR. Entropy-based feature selection with 10-fold CV is applied to a model development set to identify features most relevant for predicting AD severity. SMOTE technique is implemented to balance unbalanced disease classes in the model development set in order to avoid inflated performance estimates. 10-fold CV is used to evaluate the capability of various scoring functions of BNs and determine the BN with the optimal predictive performance. The trained BN models are evaluated on an independent test set partitioned from the original data. Prior knowledge from domain experts is used to provide constraints in structure learning (see Methods section for more details).

In this work, we apply a combination of complementary data mining and BN modelling approaches on a heterogeneous longitudinal dataset to efficiently identify key features from coarse-grained data and understand probabilistic dependencies among multiple AD factors and their changes over time. In particular, we used the Australian Imaging Biomarkers and Lifestyle flagship study of ageing (AIBL) dataset^21^, one of the largest, well-characterised, longitudinal studies on healthy ageing and AD. The analysed coarse-grained AIBL data was collected every 18 months for the period of 4.5 years. We considered the following features: (i) the ApoE allele type instead of genome sequence data; (ii) the total number of active pixels (PET) and the total volume (MRI) from brain imaging data instead of the data associated with the specific brain region; (iii) overall scores from psychological/functional tests instead of specific questions from a test; and (iv) the overall neurological history instead of information on specific neurological disorders. Compared with the Alzheimer’s Disease Neuroimaging Initiative (ADNI) data^22^, the AIBL data provides more PiB-PET data samples and more allele information of the ApoE genotype. The AIBL data is also more heterogeneous, including both imaging and non-imaging data types, than the Open Access Series of Imaging Studies (OASIS) which has only imaging data^23^. Our proposed computational framework is summarized in Fig. 1.

## Results

### Significant correlation between clinical diagnosis and clinical dementia rating

**Figure 2:**
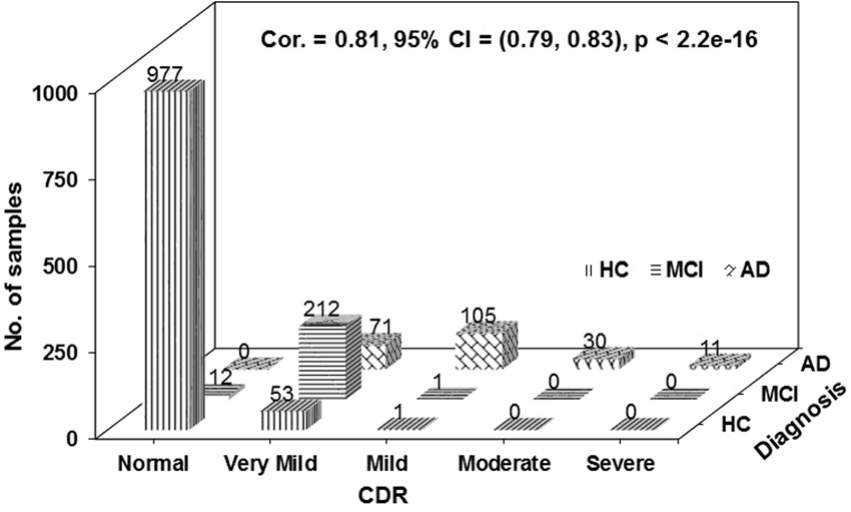
Strong correlation between clinical diagnosis and Clinical Dementia Rating (CDR) categories. Vertical bars: healthy control (HC); horizontal bars: MCI; diagonal brick bars: AD; Cor.: correlation coefficient; CI: confidence interval. CDR scores reflect 5 categories: normal controls (CDR = 0), very mild (CDR = 0.5), mild (CDR = 1), moderate (CDR = 2), and severe (CDR = 3) patients. Clinical diagnosis contains 3 categories: HC, MCI, and AD. The data distribution along with the Cor., 95% CI, and p-value showed a significant correlation between clinical diagnosis and CDR.

Clinical dementia rating (CDR) is designed to stage the severity of AD based on the state of participants in terms of memory, orientation, judgment and problem solving, community affairs, home and hobbies, and personal care^24^. CRD has been considered as a more objective assessment for AD severity due to its gold standard to classify each individual into one of the following 5 categories by corresponding CDR scores: normal control (CDR = 0), very mild (CDR = 0.5), mild (CDR = 1), moderate (CDR = 2), and severe (CDR = 3) dementia^24^. This is in contrast to relatively subjective clinical diagnosis, e.g.^25^. Most recently, it has been reported that CDR has been used to identify AD severity^26,27^. Studies in^28^ showed that CDR is well suited to serve as a comprehensive primary outcome measure for a study that will enrol subjects with early AD and follow them to more advanced stages. To avoid subjective clinical diagnosis, we used CDR as a measure of AD severity. To provide evidence to support this, we calculated the Pearson’s product-moment correlation between clinical diagnosis and CDR. Within the AIBL data, we selected 1473 complete pairs of clinical diagnosis and corresponding CDR scores (excluding patient records with missing CDR data), and found that CDR is highly correlated to diagnosis. Figure 2 illustrates the data distribution across diagnostic categories with respect to different CDR scores. The correlation coefficient (Cor.) with respect to diagnosis is 0.81 with 95% confidence interval (CI) of (0.79–0.83) and p < 2.2e-16. This justifies our use of CDR as a more objective measurement of AD severity.

### Data setup and discretisation

We considered a total of 33 heterogeneous features: 2 demographics items, 10 medical history data, 13 blood test results including ApoE genotype, 4 psychological and functional assessments (including CDR), 4 MRI and PiB-PET imaging data (see Supplementary Table S1 for the full list of the data features and descriptions). Given the CDR categories, the distribution of dementia cases in different stages of disease severity at baseline (BL) and 18 (M18), 36 (M36), and 54 (M54) months from baseline is shown in Supplementary Table S2. The data was arranged into the following 3 groups: (1) 197 participants with complete data at BL and 130 participants with complete data occurring at least once within M18-M54; (2) 133 participants with complete time-evolved data at both BL and at least once within M18-M54; and (3) 57 participants with complete time-evolved data across all times. Compared with the first 2 groups, group 3 has much smaller data samples, especially the corresponding CDR of this group has only 2 categories (i.e., normal and very mild). As this effect will be significant in the study of time evolution of relationships in the data, we shall focus on the first 2 data groups (See Groups 1 and 2 in Supplementary Table S2).

We discretised all features in order to make the subsequent BN robust and prevent over-fitting during structural learning^29^. The data features were discretised with respect to CDR categories using the class-attribute interdependence maximization (CAIM) algorithm^30^ (see Methods). The feature values were grouped into specific sets of intervals depending on how many categories each feature has. In both Groups 1 and 2, there was no subject in the severe CDR category. As the number of individuals in the moderate CDR category was relatively small, the subjects from mild and moderate CDR categories were combined into one mild/moderate CDR category. CDR and MMSE were discretised into 3 and 4 intervals, respectively, according to their definitions (Supplementary Table S1). ApoE genotypes were discretised into 5 categories as there are a total of 5 different combinations of alleles: ε3ε2, ε3ε3, ε4ε2, ε4ε3, and ε4ε4. Features without explicit categories were discretised into the same number of intervals as that of CDR i.e. 3 intervals.

### Feature selection and data balancing

For easier interpretation, shorter training time, and to prevent overfitting and reduce noisy data, feature selection is needed before data modelling. We concurrently applied entropy-based information gain, information gain ratio, and symmetrical uncertainty algorithms, in order to obtain a consistently significant subset of features. Note that the feature selection procedure was applied to the model development set only^31^. After 10-fold cross validation (10-FCV), the most relevant features were identified including three psychological/functional assessments (MMSE, LMIR, and LMDR), neuroimaging features extracted from MRI (grey matter volume (GM) and cerebrospinal fluid volume (CSF)) and active voxels from PiB-PET, ApoE genotype, and age. In total, 8 out of 32 most important and stable features with respect to CDR were selected for model training. All selected features had selection frequency of 100%. To further validate our feature selection results, we carried out the correlation analysis between CDR and the selected features. Pearson’s and Spearman’s correlation filtering showed that the three considered psychological/functional assessments were the most significant features with respect to CDR, followed by the three neuroimaging features, ApoE and age. This observation was consistent with the outputs of the three implemented entropy-based feature selection algorithms. The rankings of the features with respect to the CDR category obtained for each fold are shown in Supplementary Figures S5–S14.

### Probabilistic dependencies among key features

**Figure 3:**
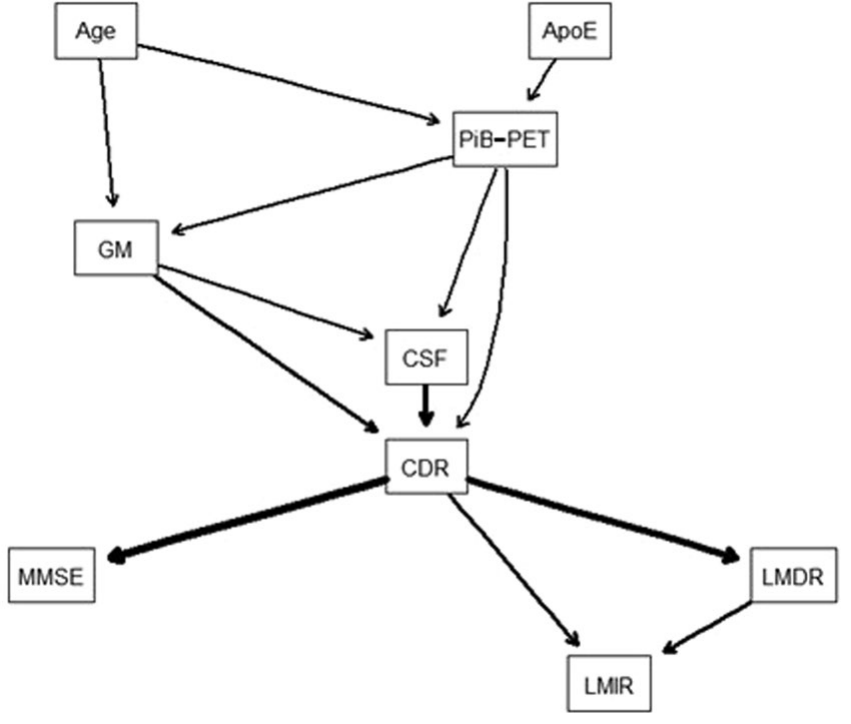
Optimized Bayesian network (BN) structure via 10-fold CV with probabilistic dependencies among predisposing factors, psychological/functional assessments, and AD severity. ApoE: apolipoprotein E genotype; GM: grey matter volume; CSF: cerebrospinal volume; PiB-PET: Pittsburgh compound B - positron-electron tomography; CDR: clinical dementia rating; MMSE: mini-mental state examination; LMIR/LMDR: logical memory immediately/delayed recall. BN is constructed based on the complete data. The thickness of the arrows represents the strength of the probabilistic influence between features. CDR is directly influenced by neuroimaging-based CSF, GM, and PiB-PET, while indirectly influenced by age and ApoE. The probabilistic influences between CDR and psychological/functional assessments are much stronger than those between predisposing indicators/biomarkers and CDR.

A total of 589 subjects with a complete data set of GM, CSF, PiB-PET, ApoE genotype, age and cognitive/functional assessments collected over the period of 4.5 years was used for BN modelling. Prior to learning, domain knowledge was identified to aid the BN structure learning process (see Methods section for more details). The optimized BN structure via 10-fold CV is shown in Fig. 3. Here, each rectangle corresponds to a domain variable while arrows denote probabilistic dependencies between associated variables. Thicker arrows reflect stronger influences between variables (determined by the p-value of the corresponding influence). Example realizations of BN structures extracted from different folds during the cross validation procedure are shown in Supplementary Figure S15. The BNs in Supplementary Figure S15 look generally similar to the BN in Fig. 3, with most of the variations coming from the weaker (less probable) connections.

Figure 3 shows that for the predisposing factors, CDR is directly influenced by CSF, followed by GM and PiB-PET, while age and ApoE indirectly influence CDR via GM, PiB-PET, and CSF. The probabilistic influences from PiB-PET to GM and CSF, and that from GM to CSF are also discovered by the BN. In terms of psychological/functional assessments, the BN reveals that the probabilistic influences between CDR and psychological/functional assessments are much stronger than those between predisposing indicators/biomarkers and CDR. CDR is most strongly linked to MMSE, followed by LMDR, then LMIR. The BN also shows the directly probabilistic dependency from LMDR to LMIR. The BN model constructed using predisposing indicators/biomarkers and their direct/indirect influences on the CDR score achieved the multi-class classification accuracy (MCA) of 0.72, 95%CI [0.59, 0.83] and AUC of 0.81.

### Dynamic changes of probabilistic dependencies in network structure

**Figure 4:**
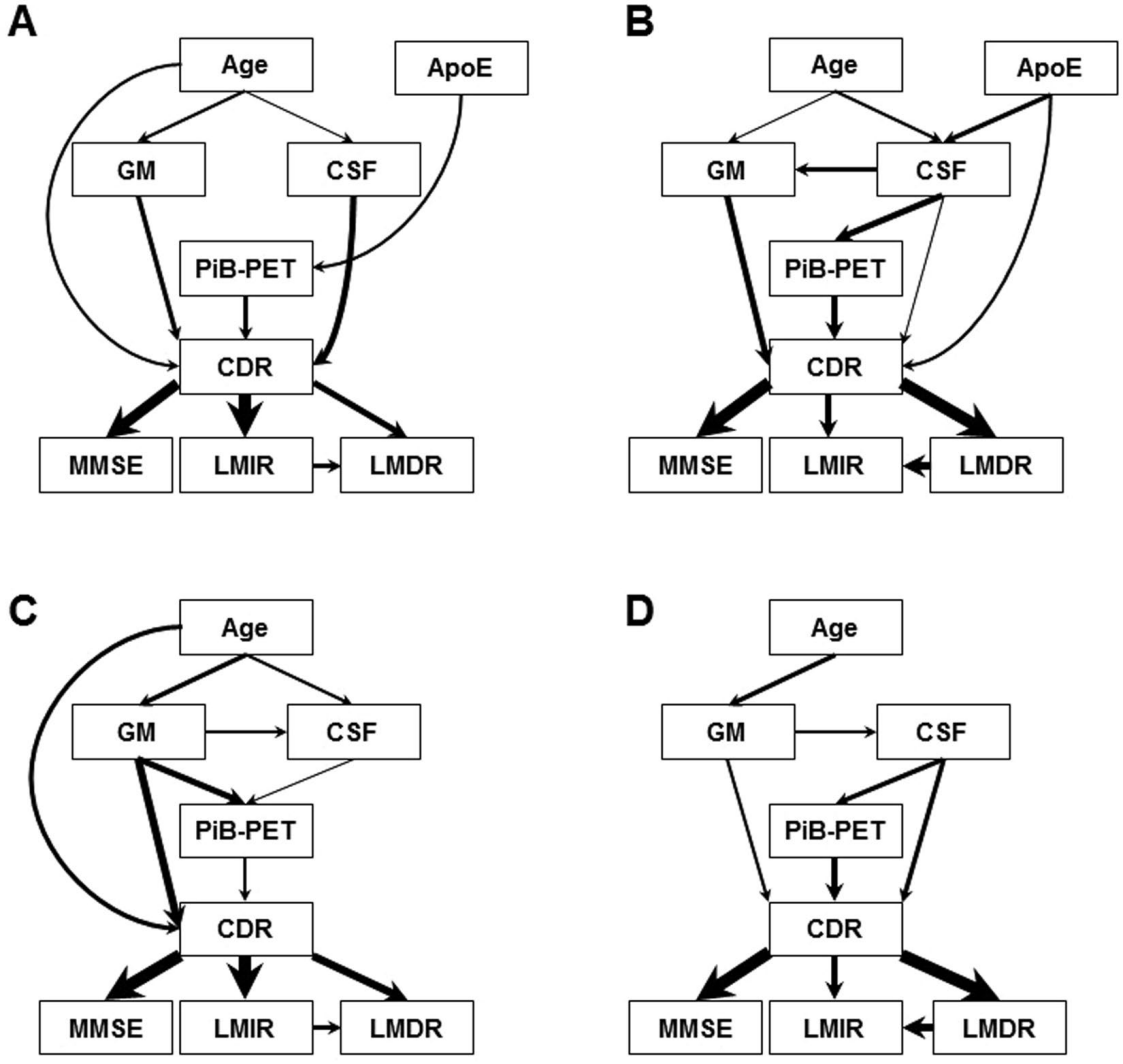
Bayesian networks based on the complete data at different times. (**A**) Group 1 data at BL (197 participants). (**B**) Group 1 data, assessments conducted at least once during the M18-54 time interval (130 participants). (**C**) Group 2 longitudinal data at BL (133 participants) including time-evolved features. (**D**) Group 2 longitudinal data, assessments conducted at least once during the M18-54 time interval including time-evolved features. The thickness of arrows represents the strength of the probabilistic influences between variables. As the Group 2 set uses longitudinal data focuses, the ApoE feature was disregarded due to its unchanging nature.

Taking advantage of the available longitudinal dataset, we constructed BNs across 2 different time points (BL and at least once at a later time during the M18-54 time interval) for two groups of participants. Group 1 consisted of 197 subjects at BL and 130 subjects assessed at least once within the M18–M54 time interval (referred to as ‘Later time’). Group 2 included the same 133 participants at both BL and the ‘Later time’. Figure 4 shows BNs constructed for Group 1 at BL (Fig. 4A), Group 1 at M18–54 (Fig. 4B), Group 2 at BL (Fig. 4C), Group 2 at M18–54 (Fig. 4D).

Given Group 1, the obtained BN at BL showed a sensitivity of 0.82 and specificity of 0.60 for healthy control, a sensitivity of 0.41 and specificity of 0.78 for very mild AD, a sensitivity of 0.33 and specificity of 0.96 for mild/moderate AD (Supplementary Table S3). The MCA and AUC for the BN model based on the Group 1 data was 0.67 and 0.80 respectively.

As the distributions of CDR categories at a later time are different from those at BL, some probabilistic dependencies at a later time may differ from those at BL. In particular, there are 6% (12/197) mild/moderate AD patients at BL compared to 12% (15/130) at a later time. Compared to the BN at BL (Fig. 4A), the BN at a later time (Fig. 4B) (MCA = 0.82, AUC = 0.81) retains most of the BN structure. However, the BN in Fig. 4B includes an indirect influence between age and CDR through CSF, GM, and PiB-PET, instead of directly influencing CDR as it does at BL. Further, CDR is now directly influenced by ApoE even stronger than by CSF. This indicates ApoE may become an important biomarker for AD severity over time. MMSE maintains a strong direct influence from CDR. The significant change from the BN at BL is the probabilistic dependencies between logical memory recall assessments and CDR, i.e., LMDR becomes more important than LMIR.

To provide further support to the dynamic changes of BNs constructed using the Group 1 data (Fig. 4A,B), we conducted the process of BN learning for Group 2 subjects, in which the same participants were assessed at both BL and ‘Later time’ (see Supplementary Table S2). Since the BN models were constructed using the same set of subjects at BL and ‘Later time’, only time-evolved features were incorporated into the models. Therefore, ApoE genotype information was not included for BN learning.

When comparing Group 2 BN models at BL (Fig. 4C) and at ‘Later time’ (Fig. 4D), we see that age did not directly influence CDR at ‘Later time’ while CSF became directly linked to CDR. In addition, CDR was influenced by PiB-PET more strongly than GM and CSF. More importantly, psychological/functional assessments showed again much stronger influence on CDR than age and imaging features. For both BNs, the direct influences on AD severity are GM and PiB-PET, which are in turn directly influenced by age and indirectly influenced by age via CSF and/or GM. This may suggest that GM, PiB-PET, and CSF could be important biomarkers for older cohorts. Overall, most of the relationships within the BNs constructed for Group 2 (Fig. 4C,D) are consistent with those developed in BNs using the Group 1 data. The differences arise only from the weaker connections, namely, GM-CSF-PiB-PET. The BNs’ MCAs/AUCs (Fig. 4C,D) at BL and ‘Later time’ are 0.74/0.89 and 0.84/0.92 respectively. The sensitivity and specificity for each AD severity category is listed in Supplementary Table S3.

### Bayesian network identified predisposing indicators and biomarkers for classification of AD severity

We now make use of the key predisposing factors and biomarkers and their relations identified by the BNs for classification of AD severity. In order to use a larger volume of data, we considered all available complete data samples regardless of time point. Supplementary Table S2 (bottom panel) lists data distribution across four AD severity categories (i.e. normal controls, very mild, mild, and moderate AD), for different combinations of predisposing factors and biomarkers. For example, we considered a total of 1480 subjects with recorded age and CDR metric; this number was reduced to 1454 records when ApoE was included in the construction of BNs.

**Figure 5:**
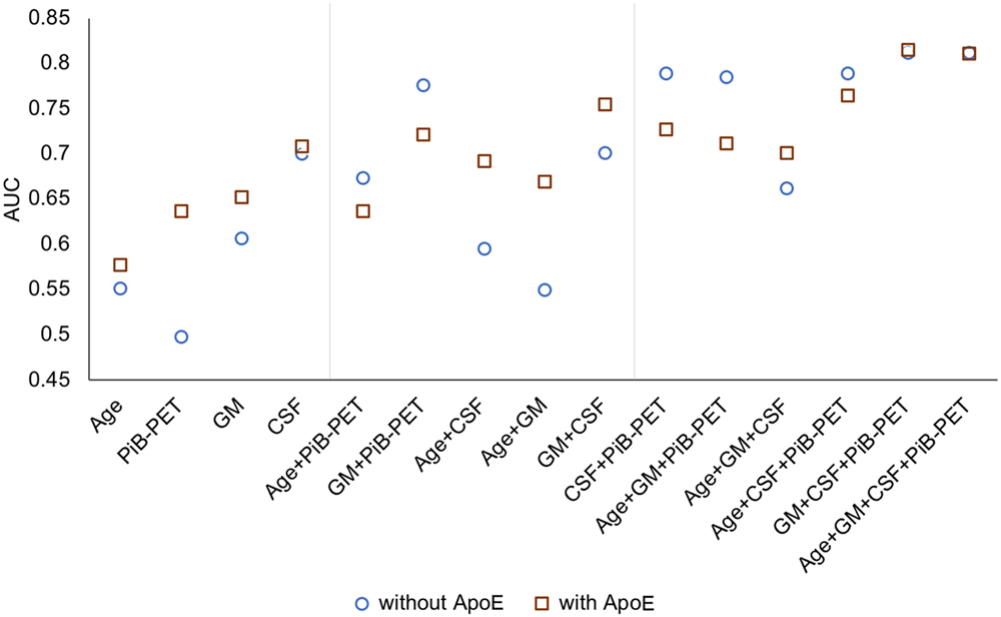
Classification accuracy (AUC) of individual predisposing indicators and biomarkers and their combinations with respect to CDR. Circled markers: BN models constructed using individual as well as combinations of predisposing factors/biomarkers without ApoE. Squared markers: BN models constructed using individual as well as combinations of predisposing factors/biomarkers with ApoE. The incorporation of ApoE into the BN structure generally improved the model performance.

Figure 5 shows the AUC values calculated for BNs constructed based on different combinations of predisposing factors/biomarkers without ApoE (circled markers) and with ApoE (squared markers). The BN models using individual markers, i.e. CSF, GM, PiB-PET, and age, provide relatively lower AUC in identifying AD severity, compared to their combinations. Only one BN model based on the combination of AD markers, specifically Age and PiB-PET (with ApoE), achieved lower AUC than BNs constructed using individual biomarkers.

The BN model incorporating all imaging biomarkers (i.e., GM + CSF + PiB-PET) with/without age was found to have the highest AUC of 0.81. These results show that using neuroimaging markers can provide sufficiently high detection of AD severity, despite the coarseness (total volume or active voxels) in the brain imaging data.

The incorporation of ApoE feature into the construction of BN models generally improved the model performance with the exception of models including both GM or PiB-PET and ApoE. The combination of imaging markers and ApoE (i.e. GM + CSF + PiB-PET + ApoE) used for BN learning showed the highest AUC of 0.82, followed by the combination of age, imaging markers, and ApoE (i.e. age + GM + CSF + PiB-PET + ApoE) with AUC of 0.81.

Finally, we constructed BN models for which the measure of AD severity was able to be directly or indirectly influenced by predisposing factors (i.e. age, GM, CSF, PiB-PET, ApoE) as well as the output of psychological/functional assessments. We specifically tested the combination of all predisposing factors with individual as well as different configurations of cognitive/functional tests (Table 1). We observed that BN models incorporating cognitive/functional assessments consistently achieved higher AUC than models based solely on predisposing factors. The best model performance was achieved for a set of age, ApoE, neuroimaging markers and all 3 functional/cognitive assessments as well as for a combination of neuroimaging markers, age, ApoE, MMSE, and LMDR. In both cases, we reported AUC of 0.91 and MCA of 0.80, 95%CI [0.67, 0.89].

**Table 1:** The AUC and MCA performance of the BN models constructed based on the combination of all predisposing factors with cognitive/functional assessments.

Features	AUC	MCA
All predisposing factors (Age + GM + CSF + PiB-PET + ApoE)	0.81	0.72, 95%CI [0.59, 0.83]
All predisposing factors + MMSE	0.86	0.76, 95%CI [0.63, 0.86]
All predisposing factors + LMIR	0.85	0.73, 95%CI [0.60, 0.84]
All predisposing factors + LMDR	0.83	0.69, 95%CI [0.54, 0.79]
All predisposing factors + MMSE + LMIR	0.89	0.81, 95%CI [0.69, 0.90]
All predisposing factors + MMSE + LMDR	0.91	0.80, 95%CI [0.67, 0.89]
All predisposing factors + LMIR + LMDR	0.87	0.75, 95%CI [0.62, 0.85]
All predisposing factors + MMSE + LMIR + LMDR	0.91	0.80, 95%CI [0.67, 0.89]

## Discussion

In this study, we successfully applied a hybrid computational approach, which includes BN based data modelling, to holistically and efficiently identify multiple important factors, the strength of their probabilistic influences and their changes in relation to AD severity (based on CDR). This was performed on the heterogeneous 4.5 years AIBL data, which consists of rapidly and easily acquired coarse-grained data of various predisposing factors, biomarkers, and psychological/functional assessment scores.

The BNs showed that age, MRI-based GM and CSF, and PiB-PET can directly influence AD severity. These 4 direct indicators, when combined, can substantially enhance the correct classification of AD severity in comparison to individual factors (AUC of 0.81), despite the data being rather coarse-grained (e.g. total brain imaging volume or active voxel). In fact, by adding the appropriate cognitive/functional assessment features, we could substantially improve the model. Hence BNs across different time points can be used as a quick and sufficiently accurate identification method of important markers of AD severity. Further, all the BNs consistently showed that psychological/functional assessments were strongly influenced by AD severity. Hence, our model supports the use of these assessments as important components of the clinical diagnostic process.

Our results are consistent with many studies on AD, including those based on the AIBL data. For instance, our BN identified the important role of CSF volume as a biomarker for detecting and predicting MCI and AD, similar to^32^. Within the AIBL study/data, ApoE ε4 allele has also been shown to be a biomarker for predicting cognitive decline over 18 months^33,34^. However, it should be emphasized that our work did not subjectively pre-select the data types and perform limited correlation evaluation. The computational approach objectively integrates and evaluates a large portion of the data, before identifying the important factors through probabilistic dependencies using BNs. The reduced number of identified factors can in turn suggest a smaller, but more efficient, number of tests for identifying or predicting very mild or mild AD.

To avoid subjective clinical classification and diagnosing of AD, we have used clinical dementia rating (CDR, which assesses 3 domains of cognition and 3 domains of function) as a more objective assessment for AD severity, namely categorizing severity into 5 categories: normal control, very mild, mild, moderate, and severe with CDR values of 0, 0.5, 1, 2, and 3, respectively^24^. Recent work^26^ has also used CDR instead of clinical diagnosis as a measure of AD severity. However the study scaled CDR into 3 categories: normal control, mild, and severe AD with CDR values of 0, 0.5, and others, and hence has a smaller range than ours while not strictly adhering to the CDR definition. Further, our work here justified CDR as an index of AD severity based on its high correlation with clinical diagnosis.

With regards to computational approaches, a BN model for MCI and AD has previously been proposed^35^. However, the work demonstrated that manually constructed BNs are simpler and more readable for physicians than those learned from data in a fully automated way. In our work, we provided a semi-automated modelling approach, in which prior knowledge was modelled manually according to domain experts and the BNs were then constructed automatically using an appropriate learning algorithm (see Fig. 1). This resulted in a more informative BN that not only revealed probabilistic relations of various factors with AD severity, but also among themselves. Other studies using BN analyses on AD were limited to the use of biomarkers^10^ or non-imaging data^36^. In comparison, our work explored a wider variety of data. A more recent study has used the structural equation (latent variable) modelling approach to identify dependencies linking brain pathology to a wide range of cognitive assessments^37,38^. However, it is not clear how the probabilistic relationships among the heterogeneous data types would likely be. The most distinctive parts of our BN modelling work were the inclusion of data types across very different levels, and the discovery of the changes in probabilistic dependencies.

Our present study can be extended in several ways. First, our modelling approach would need to be extended to handle data with missing values for larger sample size. Second, instead of using the convenient total MRI (GM, CSF, WM) volume, the MRI data can be segmented into vulnerable regions to be re-investigated. As the data size will be larger, other brain imaging modalities, such as FDG-PET, functional MRI, diffusion tensor imaging (DTI) and electroencephalography/magnetoencephalography (EEG/MEG), can also be jointly investigated. Third, it would be interesting to investigate larger datasets and with additional types of data, especially for conversion samples, e.g., the ADNI dataset.

In conclusion, we have proposed an efficient hybrid computational approach to identify key features within heterogeneous coarse-grained data with respect to Alzheimer’s disease severity. The probabilistic relationships among the identified data features can be obtained using Bayesian network modelling, with multiple Bayesian networks used to model the relationships at different times. These key data features and their relationships can then be used for disease severity classification.

## Methods

### Data description and distribution

Data was collected by the AIBL study group. AIBL study methodology has been reported previously^21^. Informed consent was obtained from all subjects. The usage of the AIBL data and our submission of the study have been approved by the AIBL Management Committee. Within the AIBL non-imaging dataset, there were a total of 861 participants at BL. However, only 262, 222, and 142 participants followed up the study after M18, M36, and M54, respectively. The data contained demographics, medical history, ApoE genotype, psychological/functional assessments, blood analyses, and clinical diagnoses. The brain imaging dataset consisted of structural MRI and PET data. We split the MRI data into 3 complementary features: grey matter (GM), white matter (WM), and cerebrospinal fluid (CSF) volumes, as they can be quickly obtained in comparison to segmented vulnerable regions. A total of 613 participants at BL had MRI scan, while only 188, 143, and 112 participants followed up the scan after M18, M36, and M54, respectively. The PET data is categorised into PiB- and FDG-PET data. Within the PiB-PET data, feature active voxels, were collected from 207, 177, 137, and 93 participants at BL, M18, M36, and M54, respectively. As the number of samples with complete FDG-PET data is very few, we exclude FDG-PET in this study. A detailed data description we considered is listed in Supplementary Table S1, with a total of 35 features involved.

Classification of MCI and AD within the cohort were made according to established, internationally recognized criteria after thorough review by a multi-disciplinary group of academic clinicians experienced in the assessment, diagnosis and management of late-life cognitive disorders, particularly MCI and AD^21^. We ignored a very small number of cases with unknown diagnosis and frontotemporal dementia (FTD). Data distribution across diagnostic categories over time is listed in Supplementary Table S4.

### Feature selection and data balancing

The feature selection process with 10-fold cross validation was applied exclusively to the model development set after setting aside a 10% (independent test set) of the complete data (see Fig. 1). Three entropy-based feature selection algorithms were used to select the most significant features with respect to clinical dementia rating (CDR). The algorithms are information gain (IG), information gain ratio (IGR), and symmetrical uncertainty (SU)^39^, which are defined respectively by

1 $$IG=H(Class)+H(Attibute)-H(Class,\,Attribute)$$

2 $$IGR=\frac{H(Class)+H(Attibute)-H(Class,\,Attribute)}{H(Attibute)}$$

3 $$SU=2\frac{H(Class)+H(Attibute)-H(Class,\,Attribute)}{H(Attibute)+H(Class)}$$

where *H* is Shannon’s Entropy defined by $H(X)=-{\sum }_{i=1}^{n}P({X}_{i}){\mathrm{log}}_{2}P({X}_{i})$, with *P* as the probability function^40^. These were used to find weights of discrete attributes based on their correlation with the target classes. The advantage of the entropy filter is that it makes no assumptions about the nature of the data and no disturbances occurring in dynamic environments. The technique demonstrated its effectiveness in a range of applications e.g. in gene selection for cancer classification^41^.

It has been shown that the class imbalance in a data set introduces a bias in the performance of predictive models due to their preference towards the majority class^42^. We therefore balanced the unbalanced disease classes in the model development set by resampling the original data and creating synthetic instances using the synthetic minority oversampling technique (SMOTE)^43^.

### Data discretization

The class-attribute interdependence maximization (CAIM) algorithm^30^ was applied to discretize data features with respect to CDR categories. The CAIM criterion measures the dependency between the class variable and the discretization variable for attribute, and is defined as:

4 $$CAIM=\frac{\sum _{r=1}^{n}\frac{ma{x}_{r}^{2}}{{M}_{+r}}}{n}$$

for $r=1,\,2,\,\ldots ,\,n$, in which $ma{x}_{r}$ is the maximum value within the *r*^th^ column of the quanta matrix (also called a contingency table^30^). ${M}_{+r}$ is the total number of continuous values of the attribute that are within the interval.

### Bayesian network modelling

Bayesian networks (BN) were implemented to provide a representation of probabilistic dependencies within the heterogenous AD data using directed acyclic graphs. The nodes in the BN corresponded to the domain variables and the arcs reflected the probabilistic dependencies between associated variables^44^. Given a set of *n* variables, $X=\{{X}_{1},{X}_{2},\,\cdots ,{X}_{n}\}$, a BN represents a joint probability distribution on $X,$ $P(X)$, defined as

5 $$P(X)=\prod _{{X}_{i}\in X}P({X}_{i}|pa({X}_{i})),\,i=1,\,2,\,\cdots ,\,n$$

where $pa({X}_{i})$ is the set of parents of ${X}_{i}$.

In order to construct BN structures that reflect probabilistic dependencies in the real data, we applied a combination of prior knowledge and data-oriented modelling. We first used expert knowledge, including current diagnostic criteria and input from physicians, to select relevant AD indicators/markers and then, identified causal and forbidden relationships among variables as structural constraints for BN learning. This procedure allowed us to search for optimal network structures over a restricted topological space and hence, significantly improve computational efficiency. Note that the constraints were not exhaustive i.e. there were multiple BNs that adhered to a given set of constraints. Accordingly, predisposing indicators/biomarkers were presumed to influence directly/indirectly the measure of AD severity, which in turn could affect the output of psychological/functional assessments. Neuroimaging factors could influence each other, as well as psychological/functional assessments. Once relevant constraints were identified, a score-based algorithm was implemented for learning the structures of a BN. The Hill Climbing (HC) score-based learning technique was used to identify high-scoring network structures by evaluating local changes to a potential network solution and selecting the one that maximized the score function^45^. The following scoring functions were tested: K2 score^46^, Bayesian Dirichlet equivalent (BDE) score^47^, modified Bayesian Dirichlet equivalent (MBDE) score^47^, and Bayesian Information Criterion (BIC) metric^48^.

In order to evaluate the capability of various fitting functions of BNs and compare the performance of different BN structures, we applied the 10-fold cross validation (CV) procedure^49^. Given a model development set (see Fig. 1), we randomly partitioned the data into k = 10 subsets. Each subset (validation set) was used in turn to validate the model fitted on the remaining k − 1 subsets (training set). The log-likelihood loss of the validation set for each BN fitted from the training set was computed. Loss estimates of each of the k subsets were then combined to determine an overall loss and to identify the optimal scoring criterion.

The generalizability of trained BN models was evaluated on an independent test set partitioned from the original data (10% of the complete dataset). Given a smaller set of longitudinal data, we used 30% of the original data for the unseen test set to obtain more reliable estimation on the testing accuracy of BN models. The criteria retained for comparison of BN models were: the area under the curve of the receiving operator characteristics curve (AUC), multi-class classification accuracy (MAC), sensitivity and specificity, all reported on the independent test set^50^.

### Hardware and software

All computations, including data pre-processing, BN construction and validation, and visualisation, were performed using the R statistical software, version 1.0.136 47 (R Foundation for Statistical Computing, Vienna, Austria)^51^.

## Electronic supplementary material

Supplementary Information
