# Supplementary material for: A hybrid computational approach for efficient Alzheimer’s disease classification based on heterogeneous data
Source: Sci Rep. 2018 Jun 27;8:9774. doi: 10.1038/s41598-018-27997-8 (PMC6021389; doi:10.1038/s41598-018-27997-8)
Supplement: Supplementary file 1 — Supplementary Information [file 41598_2018_27997_MOESM1_ESM.docx]

**Supplementary Information**

**A hybrid computational approach for efficient Alzheimer’s disease classification based on heterogeneous data**

**Xuemei Ding, Magda Bucholc, Haiying Wang, David H. Glass, Hui Wang, Dave H. Clarke , Anthony John Bjourson, Le Roy C Dowey, Maurice O’Kane, Girijesh Prasad, Liam Maguire, KongFatt Wong-Lin**

| **Category** | | **Description** |
| --- | --- | --- |
| **non-imaging data** | Demographics | 1) age: 55~96 years  2) gender: Female/Male |
|  | Medical history | 3) psychiatric (MH_PSYCH)  4) neurologic (MH_NEURL)  5) cardiovascular (MH_CARD)  6) hepatic (MH_HEPAT)  7) musculoskeletal (MH_MUSCL)  8) endocrine-metabolic (MH_ENDO)  9) gastrointestinal (MH_GAST)  10) renal-genitourinary (MH_RENA)  11) smoking (MH_SMOK)  12) malignancy (MH_MALI). Each medical history is a binary feature (i.e., Y/N) |
|  | ApoE genotypes | 13) 2 alleles genotype. Each allele holds one of three genotypes: ε2, ε3, ε4 |
|  | Neuropsychology assessments | 14) clinical dementia rating (CDR)  5 categories: healthy (0), very mild dementia (0.5), mild (1), moderate (2), and severe (3)  15) mini-mental state exam (MMSE): 0-30  4 categories: may be normal (30-25), mild/early (24-21), moderate (20-10), and severe (9-0)  16) total number of story units recalled - logical memory immediate recall (LMIR): 0~25  17) total number of story units recalled - logical memory delayed recall (LMDR): 0~25 |
|  | Blood analyses | 18) thyroid stim. Hormone (AXT117)  19) vitamin B12 (BAT126)  20) red blood cell (HMT3)  21) white blood cell (HMT7)  22) platelets (HMT13)  23) haemoglobin (HMT40)  24) mean corpuscular haemoglobin (HMT100)  25) mean corpuscular haemoglobin concentration (HMT102)  26) urea nitrogen (RCT6)  27) serum glucose (RCT11)  28) cholesterol (high performance) (RCT120)  29) creatinine (rate blanked) (RCT329) |
|  | Diagnosis | 30) diagnostic results: 3 categories, i.e., healthy control (HC), mild cognitive impairment (MCI), and Alzheimer’s disease (AD) |
| **imaging data** | MRI | 31) grey matter (GM) volume  32) white matter (WM) volume  33) cerebrospinal fluid (CSF) volume |
|  | PET | 34) active voxels in PET with Pittsburgh compound B (PiB-PET)  35) active voxels in PET with fluorodeoxyglucose (FDG-PET) |

**Supplementary Table S1. Description of the AIBL data.** FDG-PET is not used in this study due to small sample size.

| **Time and/or feature(s)** | | **AD severity (CDR category)** | | | | | |
| --- | --- | --- | --- | --- | --- | --- | --- |
|  |  | Normal (0) | Very Mild (0.5) | Mild (1) | Moderate (2) | Size |  |
| BL | | 127 | 58 | 10 | 2 | 197 |  |
| Later | M18 | 123 | 36 | 11 | 0 | 170 |  |
|  | M36 | 97 | 26 | 6 | 2 | 131 |  |
|  | M54 | 69 | 14 | 6 | 2 | 91 |  |
| Group 1 | BL | 127 | 58 | 10 | 2 | 197 |  |
|  | Later | 88 | 27 | 14 | 1 | 130 |  |
| Group 2 | BL | 95 | 34 | 4 | 0 | 133 |  |
|  | Later | 91 | 27 | 14 | 1 | 133 |  |
| Available features at all times combined | Age | 988 | 339 | 111 | 42 | 1480 |  |
|  | Age+ApoE | 976 | 328 | 108 | 42 | 1454 |  |
|  | Age+GM+CSF | 758 | 218 | 59 | 18 | 1053 |  |
|  | Age+GM+CSF+ApoE | 737 | 210 | 56 | 18 | 1021 |  |
|  | Age+GM+CSF+PiB-PET | 428 | 136 | 37 | 11 | 612 |  |
|  | Age+GM+CSF+PiB-PET+ApoE | 428 | 136 | 35 | 11 | 610 |  |

**Supplementary Table S2.** **Complete data distribution across CDR categories over different times.** Data is distributed according to data features and their combinations across CDR categories over different times. BL, M18, M36, M54: data at baseline, and months 18, 36 and 54, respectively. Later time: from M18 onwards.

| **Data group** | **Time** | **AD severity (CDR category)** | | | | | | **All CDR categories** | | | |
| --- | --- | --- | --- | --- | --- | --- | --- | --- | --- | --- | --- |
|  |  | Normal (0) | | Very Mild (0.5) | | Mild/Moderate  (1 or 2) | | Accuracy | Kappa | 95% CI | AUC |
|  |  | Sen. | Spe. | Sen. | Spe. | Sen. | Spe. |  |  |  |  |
| 1 | BL | 0.82 | 0.60 | 0.41 | 0.78 | 0.33 | 0.96 | 0.67 | 0.31 | 0.54-0.79 | 0.80 |
|  | Later | 0.88 | 0.75 | 0.63 | 0.90 | 0.75 | 0.97 | 0.82 | 0.61 | 0.66-0.92 | 0.81 |
| 2 | BL | 0.82 | 0.63 | 0.50 | 0.83 | 1.00 | 0.97 | 0.74 | 0.41 | 0.58-0.87 | 0.89 |
|  | Later | 0.89 | 0.83 | 0.63 | 0.90 | 1.00 | 0.97 | 0.84 | 0.68 | 0.70-0.94 | 0.92 |

**Supplementary Table S3. The BN classification accuracy for each CDR category and the whole classification accuracy for all CDR categories.** Sen.: sensitivity, Spe.: specificity. Group 1: 197 participants with complete data at BL and 130 participants with complete data occurring at least once within M18-M54 (later time). Group 2: 133 same participants with complete time-evolved data at both BL and at later time.

| **Time** | **Non-imaging data** | | | | |  | **Brain scanned imaging data** | | | | | | | | | | |
| --- | --- | --- | --- | --- | --- | --- | --- | --- | --- | --- | --- | --- | --- | --- | --- | --- | --- |
|  |  |  |  |  |  |  | MRI scanned data | | | | | | PiB-PET scanned data | | | | |
|  | HC | MCI* | AD | NA | FTD | Size | HC | MCI | AD | NA | FTD | Size | HC | MCI | AD | NA | Size |
| BL | 609 | 143 | 105 | 1 | 3 | 861 | 456 | 91 | 63 | 1 | 2 | 613 | 141 | 39 | 27 | 0 | 207 |
| M18 | 172 | 34 | 55 | 1 | 0 | 262 | 141 | 21 | 25 | 1 | 0 | 188 | 134 | 20 | 22 | 1 | 177 |
| M36 | 144 | 30 | 46 | 2 | 0 | 222 | 108 | 18 | 16 | 1 | 0 | 143 | 103 | 18 | 15 | 1 | 137 |
| M54 | 108 | 18 | 15 | 1 | 0 | 142 | 87 | 12 | 12 | 1 | 0 | 112 | 70 | 10 | 12 | 1 | 93 |

**Supplementary Table S4. Data distribution across diagnostic categories over time.** HC: healthy control; MCI: mild cognitive impairment; AD: Alzheimer’s disease; FTD: frontotemporal dementia; NA: not available, i.e., diagnostic results is unknown.


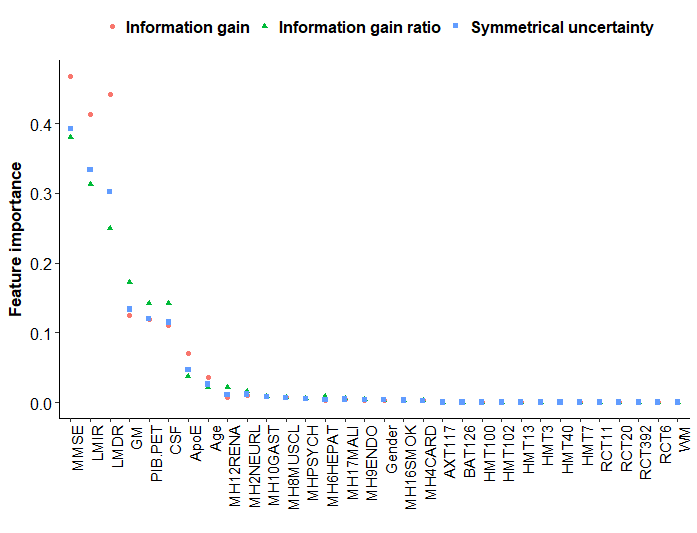


**Supplementary Figure S5**. **Features’ importance ranking with respect to CDR using 3 entropy-based feature selection algorithms on training subset of fold 1.**


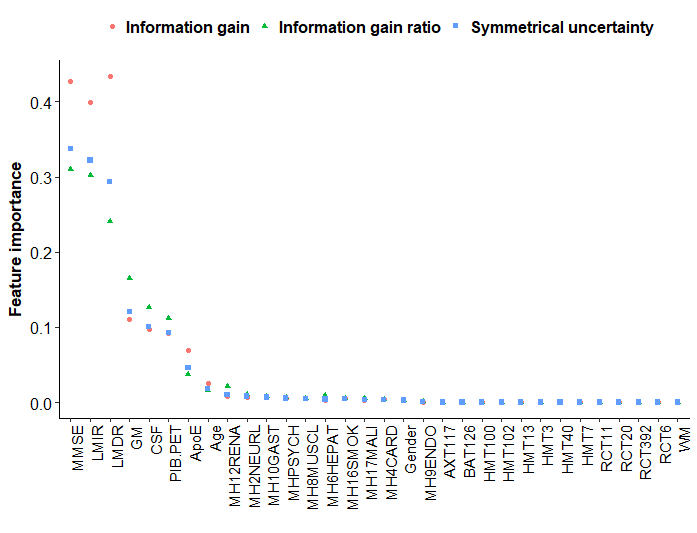


**Supplementary Figure S6**. **Features’ importance ranking with respect to CDR using 3 entropy-based feature selection algorithms on training subset of fold 2.**


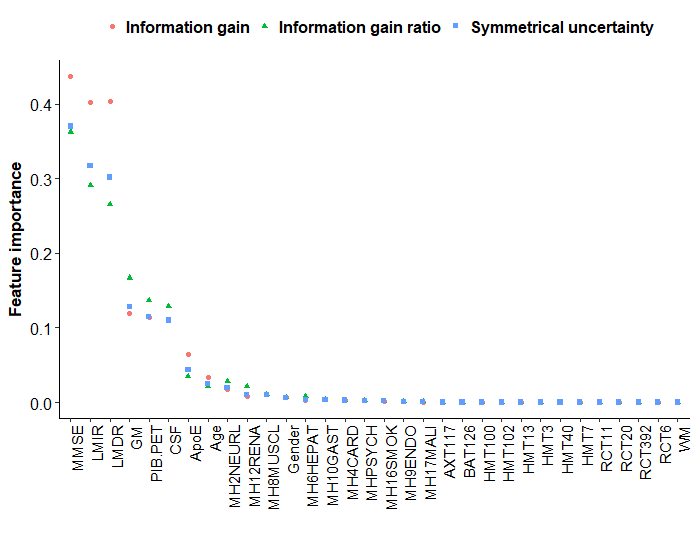


**Supplementary Figure S7. Features’ importance ranking with respect to CDR using 3 entropy-based feature selection algorithms on training subset of fold 3.**


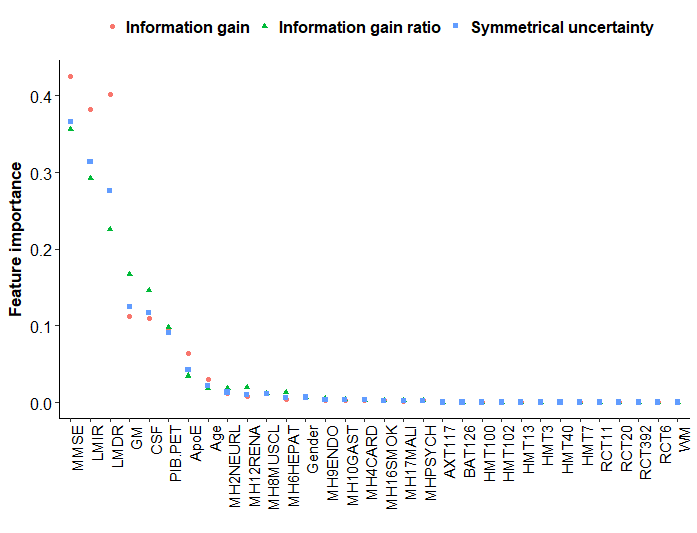


**Supplementary Figure S8. Features’ importance ranking with respect to CDR using 3 entropy-based feature selection algorithms on training subset of fold 4.**


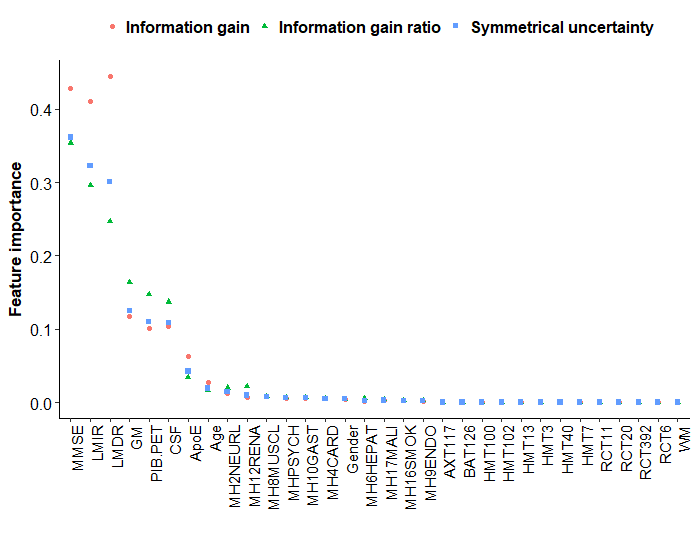


**Supplementary Figure S9**. **Features’ importance ranking with respect to CDR using 3 entropy-based feature selection algorithms on training subset of fold 5.**


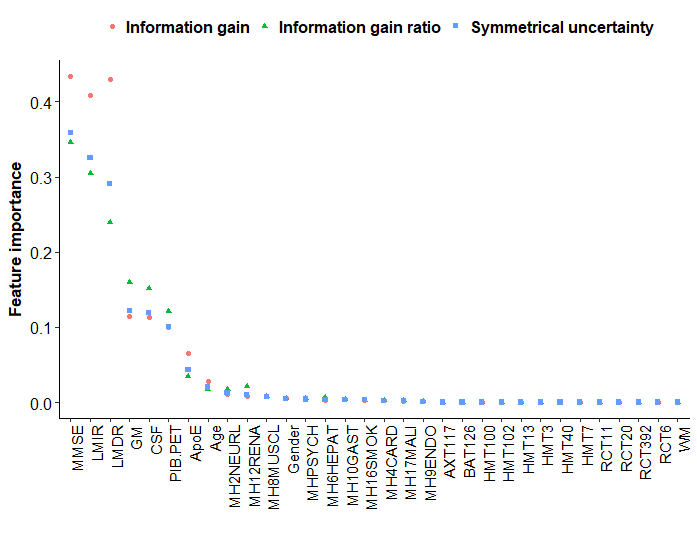


**Supplementary Figure S10. Features’ importance ranking with respect to CDR using 3 entropy-based feature selection algorithms on training subset of fold 6.**


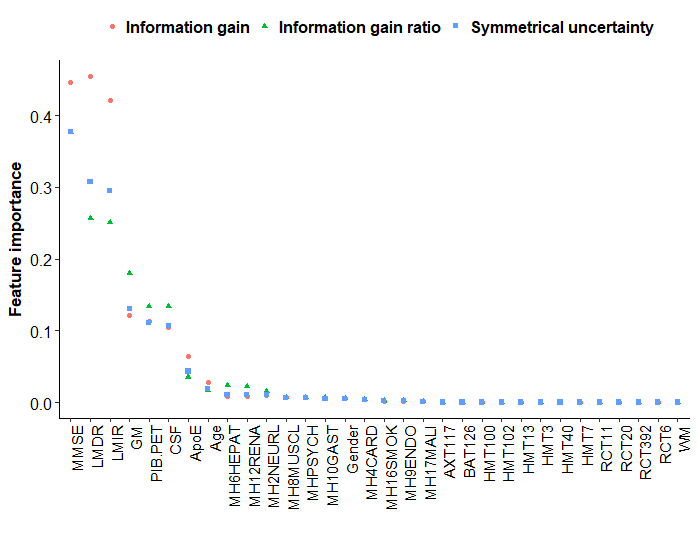


**Supplementary Figure S11**. **Features’ importance ranking with respect to CDR using 3 entropy-based feature selection algorithms on training subset of fold 7.**


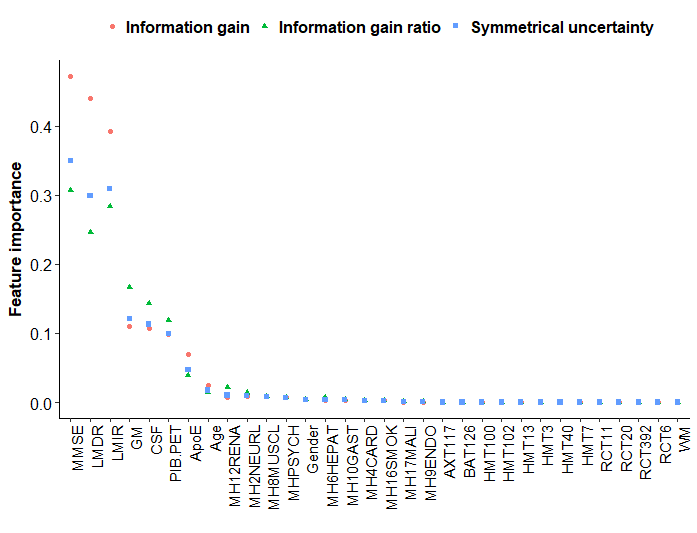


**Supplementary Figure S12**. **Features’ importance ranking with respect to CDR using 3 entropy-based feature selection algorithms on training subset of fold 8.**


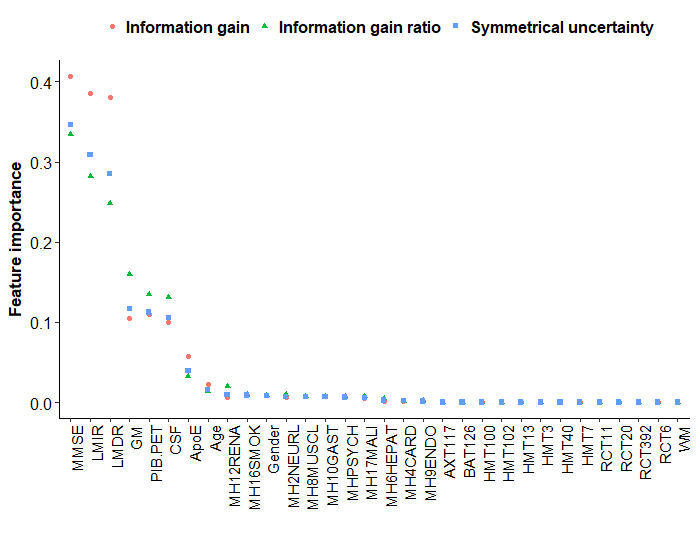


**Supplementary Figure S13. Features’ importance ranking with respect to CDR using 3 entropy-based feature selection algorithms on training subset of fold 9.**


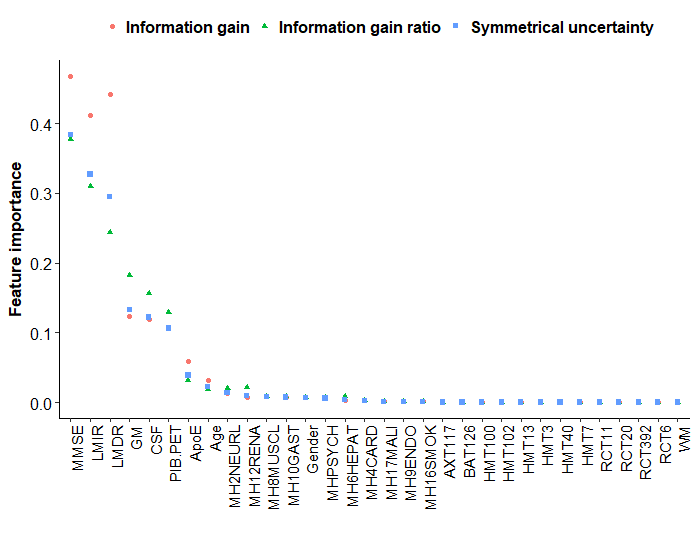


**Supplementary Figure S14. Features’ importance ranking with respect to CDR using 3 entropy-based feature selection algorithms on training subset of fold 10.**

**
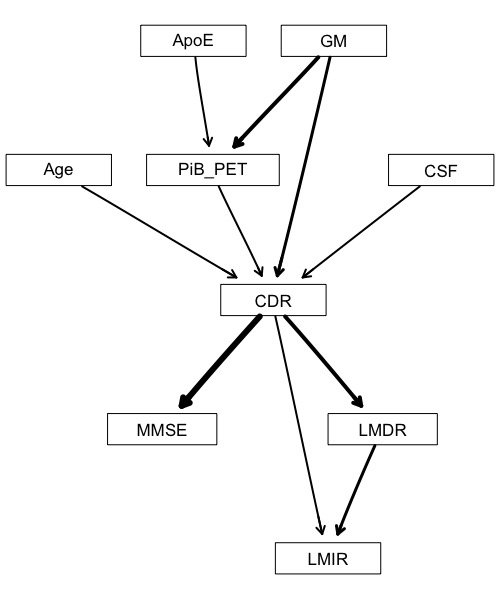
**

**(a)**

**
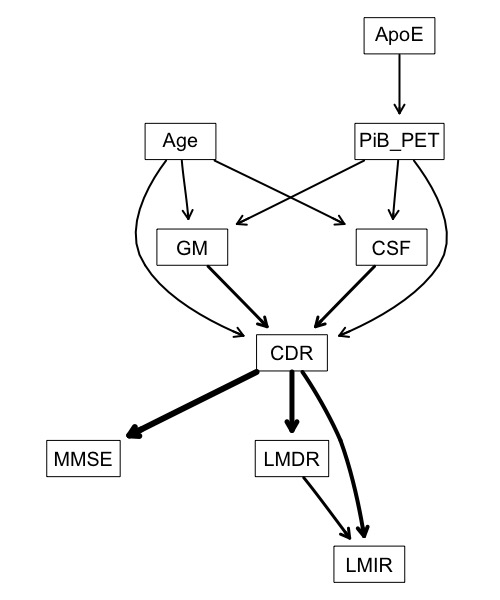
**

**(b)**

**
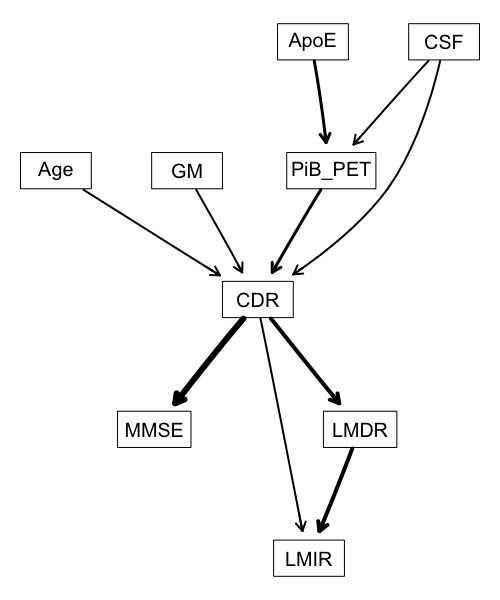
**

**(c)**

**
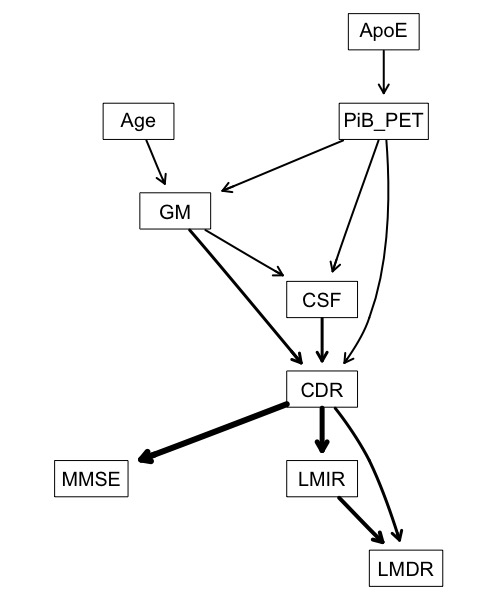
**

**(d)**

**Supplementary Figure S15. Samples of probabilistic dependencies among predisposing factors, psychological/functional assessments, and AD severity in BN structures extracted from different folds during the cross validation procedure (a, b, c, and d). AUC of (a-d) are 0.76, 0.72, 0.78, and 0.79 respectively. The optimal BN (with AUC of 0.82) structure further tested on unseen AD data is shown in Fig. 3.**
